# The Interactions between Arbuscular Mycorrhizal Fungi and *Trichoderma longibrachiatum* Enhance Maize Growth and Modulate Root Metabolome under Increasing Soil Salinity

**DOI:** 10.3390/microorganisms10051042

**Published:** 2022-05-17

**Authors:** Rong Yang, Zefeng Qin, Jingjing Wang, Xiaoxia Zhang, Song Xu, Wei Zhao, Zhiyong Huang

**Affiliations:** 1 Tianjin Institute of Industrial Biotechnology, Chinese Academy of Sciences, Tianjin 300308, China; yangr@tib.cas.cn (R.Y.); wang_jj@tib.cas.cn (J.W.); zhang_xx@tib.cas.cn (X.Z.); xu_s@tib.cas.cn (S.X.); zhao_w@tib.cas.cn (W.Z.); 2 College of Resource and Environmental Sciences, China Agricultural University, Beijing 100193, China; qzf505966460@163.com

**Keywords:** arbuscular mycorrhizal fungi, *Trichoderma longibrachiatum*, salt stress, maize, dual inoculation, metabolites

## Abstract

*Trichoderma longibrachiatum* sp. are free-living filamentous fungi which are common in agro-ecosystems. However, few studies thus far have examined the interaction between *Trichoderma longibrachiatum* and arbuscular mycorrhizal (AM) fungi in saline soil and their potential for improving plant stress tolerance. Here, single, dual-inoculated (*T. longibrachiatum* MF, AM fungal community or *Glomus* sp.), and non-inoculated maize (*Zea may* L.) were subjected to different salinity levels (0, 75, 150, and 225 mM NaCl) to test the synergistic effects of dual inoculants on maize plants in different salt stress conditions. Plant performance and metabolic profiles were compared to find the molecular mechanisms underlying plant protection against salt stress. The first experiment revealed that dual inoculation of an AM fungal community and *T. longibrachiatum* MF improved the biomass and K^+^/Na^+^ ratio in maize under non-saline conditions, and generally enhanced AM fungal growth in root and soil under all but the 225 mM NaCl conditions. However, MF inoculant did not influence the structure of AM fungal communities in maize roots. In the second experiment, dual inoculation of *Glomus* sp. and *T. longibrachiatum* MF increased maize plant biomass, K^+^/Na^+^ ratio, and AM fungal growth in root and soil significantly at both 0 and 75 mM NaCl conditions. We identified metabolic compounds differentially accumulated in dual-inoculated maize that may underline their enhanced maize plant tolerance to increasing soil salinity. Our data suggested that the combination of *Glomus* sp. and *T.* *longibrachiatum* leads to interactions, which may play a potential role in alleviating the stress and improve crop productivity in salt-affected soils.

## 1. Introduction

In agricultural settings, high levels of salinity are detrimental to crop growth. Particularly, in arid and semi-arid regions, many areas are affected by soil salinization, substantially inhibiting the productivity of crops [1]. Several agricultural engineering methods have been used to reduce the negative effects of saline-alkali land on crop productions by freshwater irrigation, organic and inorganic compound fertilizer modification, and even breeding projects. However, these approaches are costly while providing relatively minor beneficial effects. Complementary biological approaches are expected to improve crop production under saline conditions.

The plant growth-promoting microorganisms (PGPM) in agricultural soil present a potential biological way to promote plant growth and nutrient absorption under environmentally stressed conditions [2,3]. Agro-ecosystems consist of complex interaction networks among numerous microorganisms [4]. The outcome of different PGPM interactions such as these is of fundamental importance to crop growth and resistance in different agricultural fields [5], including salt-affected soils [6]. For example, Ghaffari et al. [7] found that the Basidiomycete *Piriformospora indica* can colonize a wide range of agricultural crops, increasing grain yield, and protecting their hosts from a wide range of salt levels. Recently, an isolate of endophytic fungi was identified as a key symbiont helping the host plants thrive under salt stress conditions [8]. The PGPM with a high salt resistance could potentially enhance the salt tolerance of the host plant by producing secondary metabolites [9]. As beneficial soil microorganisms used in current agricultural practices are deleteriously affected by excessive salinity, the use of salt-tolerant PGPM to enhance crop salt tolerance presents a possible novel approach to improving the resistance or increasing the crop yield in saline-alkaline soil.

*Trichoderma* spp. are free-living filamentous fungi that are common in agro-ecosystems, and some of them are the most potent agents for the biocontrol of soil borne plant pathogens [10]. Root colonization by *Trichoderma* spp. frequently enhances root growth and development, crop productivity, and the uptake and use of nutrients [11]. Recently, researchers working in this area have focused their attentions on the dual inoculation of *Trichoderma* spp. and other beneficial soil microorganisms for enhancing plant performance [9]. For example, a dual inoculation of *Trichoderma asperellum* and AM fungi increased biomass yield of plants in unfavorable environments including toxic metal-polluted agricultural areas, although *Trichoderma asperellum* does not influence the AM fungal abundance in plant roots [12]. The positive effects on plant growth and promotion of stress tolerance by synergistic interactions of beneficial soil microorganisms under hostile environments have been extensively reviewed by Nadeem et al. [13]. These microbes are believed to act as essential bio-ameliorators of stress by regulating nutritional and ionic balance [14], and inducing system tolerance to stress [15].

Among these beneficial microbes, Arbuscular mycorrhizal (AM) fungi are obligate plant-root symbionts which produce an extensive mycelial network in the soil. AM fungi can alleviate the detrimental effects of salinity on plants by assisting with the absorption of nutrients, maintaining the plants ionic balance, protecting plant enzyme activity, and facilitating the plant’s absorption of water [16]. These processes result in a lower accumulation of sodium and chlorine and thereby stabilize the plant’s K^+^/Na^+^ ratio under high saline conditions [17]. AM fungi can interact with PGPM in many ways to enhance a plant’s tolerance to environmental stress [18]. For example, Toro et al. [19] found that dual inoculation with AM fungi and PGPM increased plant growth and P uptake. Dual inoculation with AM fungi and saprophytic fungi has also been found to be an efficient bioremediation strategy for contaminated soil [20]. The combined application of AM fungi and *Trichoderma* spp. can also increase soil-borne disease suppression [21]. Moreover, neutral [21], synergistic [22], or even antagonistic [23] interactions between AM fungi and PGPM have been observed. Despite these beneficial associations of microbes, studies examining the interactions of *T. longibrachiatum* and AM fungi in saline environmental conditions are limited, especially where competition for nutrient and niches in the rhizosphere is high. This knowledge is important for our understanding of the relationship between *T. longibrachiatum* and AM fungi and their potential effect on plant stress tolerance, and for the development of crop management practices under saline environmental conditions.

Salt-affected soils contain high levels of soluble salts, predominantly NaCl, which decreases plant growth by inducing osmotic stress, nutrient uptake imbalances, and modification of a plants’ metabolic processes [24]. The former study found that AM inoculants appear as a valuable tool for stimulating a plant’s capacity to adapt their metabolism to challenging conditions, by increasing its metabolic capacity to overcome the adverse environmental stress [1]. Rivero et al. [25] reported that AM plants can perform better than nonmycorrhizal plants through markedly accumulated compounds in roots in response to salinity stress. Regarding these beneficial associations of microbes, a better understanding of how their interactions increase plant tolerance against salinity stress is fundamental. Untargeted metabolomic approaches have proved to be an excellent tool for providing a global view of plant responses under stress [26]. In particular, the LC-MS technique is highly sensitive, allowing for the detection of key molecules in plant adaptation responses.

The aim of this study was to examine the interaction between AM fungi and *T. longibrachiatum* in saline soil, and to identify the metabolites mediating such effects. We conducted two microcosm experiments, including *T. longibrachiatum* MF together with an AM fungal community or *Glomus sp*. We hypothesized that (1) *T. longibrachiatum* MF inoculant improves AM fungi growth under salt stress; (2) dual inoculation outperforms single inoculation under salt stress; (3) dual inoculation alters plants’ metabolites and mediates such effects.

## 2. Materials and Methods

### 2.1. Preparation of the T. longibrachiatum MF Inoculant

The *T. longibrachiatum* MF was isolated from the soil of a maize plant growing in salt contaminated soil, which had acclimatized to saline conditions prior. The *T. longibrachiatum* MF had been identified in a former study (NCBI accession number: MG745304) [27] and preserved at the Tianjin Institute of Industrial Biotechnology, CAS, China. The salt tolerance of MF was tested qualitatively by inoculating MF isolates on PDA plates with different NaCl concentrations (0, 75, 150, and 225 mM NaCl). The plates were sealed and incubated at 30 °C for 4 days. The growth of MF on the PDA plates with 0, 75, 150, and 225 mM NaCl indicated the salt tolerance of MF (Figure S1).

In this study, *T. longibrachiatum* MF were recovered on PDA plates incubated at 30 °C for 7 days. The spores were transferred into a liquid PDA culture and incubated at 30 °C, at 180 rpm for 5 days. The culture was centrifuged (5000× *g*, 20 min), washed in sterile deionized water, and then re-suspended in sterile deionized water to produce an inoculum of 10^6^ colony forming units (CFU)·mL^−1^ [28].

### 2.2. Preparation of the Soil

The experimental soil was collected from an agricultural region of Xinjiang province, China (44°18′ N, 86°22′ E, Changji Autonomous Prefecture) at a depth of 0–30 cm. The climate conditions in the sampling site have been described in a former study [29]. Fresh soil was selected and divided into two parts. A soil sub-sample was stored at 4 °C for the AM fungal community inoculant and another sample was air-dried and passed through a 5-cm mesh-sieve. Initial soil properties were as follows: pH 7.4 (1:2.5, soil: water, *w*/*v*), electrical conductivity 0.5 dS m^−1^ (1:5 soil: water, *w*/*v*), soil organic matter 2.5%, available nitrogen 32.7 mg·kg^−1^, available phosphorus 53.2 mg·kg^−1^, and available potassium 130.6 mg·kg^−1^.

### 2.3. Microcosm Experiment

#### 2.3.1. Experiment 1

The first experiment involved soil microcosms inoculated with AM fungal communities. The experiment was conducted in a 2 × 4 factorial design. There were two inoculated treatments (inoculated with MF and un-inoculated as a control) and four salinity levels (0, 75, 150, and 225 mM NaCl). Each of the eight treatment combinations were replicated three times to produce a total of 24 pots.

AM fungal community was isolated from the fresh soil above. AM fungal community was extracted by wet sieving, decanting, and a sucrose density gradient centrifugation as described by Utobo [30]. The AM fungal spores were collected and propagated on maize (*Zea may* L.) for 6 months in pots containing a sterilized mixture of quartz-sand and soil selected above (1:1). Finally, the substrate, spores, mycelium, and infected root segments were utilized as the AM fungal community inoculant. Approximately 10 spores were counted per gram of the AM fungal community inoculant.

Each pot contained 600 g of sterilized soil substrate mixture (3:1, soil: quartz-sand). All pots were given 100 g of AM fungal community inoculant, and AM inoculant was mixed with pot soil before seeding. Maize seeds were prepared as described in Yang et al. [29]. Three seeds were planted in each pot which, after emergence, were thinned to one seedling per pot. For the *T. longibrachiatum* MF inoculated treatment, 70 mL (10^6^ CFU mL^−1^) of conidial suspension was inoculated one day after two weeks of maize growth. Pots without MF inoculant were supplemented with 70 mL sterile water. Then, four different salinity treatments were applied over an additional two weeks. In each case, the maize plants were watered once every two days with 60 mL of different concentrations of NaCl solution (0 (sterilized water), 75, 150, or 225 mM NaCl), as described in Rivero et al. [25]. Afterward, the maize plants were grown for an additional seven weeks. The plants were grown in a controlled growth chamber for 11 weeks in total (30/24 °C day/night, 16 h photoperiod, 800 mmol m^−2^s^−1^ photosynthetically active radiation, 65% relative humidity). Pots were placed randomly, and their positions were re-arranged on a weekly basis. Each pot received a dose of 70 mL of P-free Hoagland’s nutrient solution once a week as described by Yang et al. [29].

#### 2.3.2. Experiment 2

The experiment was conducted in a 4 × 2 factorial design. There were four inoculated treatments (inoculated with *T. longibrachiatum* (MF), inoculated with *Glomus* sp. (Gm), dual-inoculated (Gm + MF), and un-inoculated as a control), and two salinity levels (0 and 75 mM NaCl). Each of the eight treatment combinations was replicated six times to give a total 48 pots.

*Glomus* sp. (Gm) was supplied by the Chinese Bank of the Glomeromycota (BGC), and they were propagated on maize for 6 months in pots containing a sterilized mixture of quartz-sand and soil described above (1:1). Approximately 10 spores were counted per gram of the *Glomus* sp. (Gm) inoculant.

Each pot was filled with 600 g of sterilized soil substrate mixture (3:1, soil:quartz-sand). Gm and dual-inoculated treatments were performed by adding to 100 g of Gm inoculant before seeding, the other pots received the 100 g of sterilized Gm inoculant to homogenize soil nutrition. Maize seeds were planted in each pot using the same methods as in experiment 1 above. MF inoculated treatments used the same methods as in experiment 1 above. Then, two different salinity treatments (0 and 75 mM NaCl) were applied as in experiment 1 above. Afterward, the maize plants were grown for an additional eight weeks. The plants were grown in a controlled growth chamber for 12 weeks in total. The condition of chamber was the same as in experiment 1 above.

### 2.4. Harvest and Sample Preparation

At harvest, maize plants were harvested carefully, and shoots were weighed and oven-dried at 105 °C for 1 h, and then at 75 °C for 48 h to determine dry weight and inorganic ion concentrations. Maize roots were washed thoroughly in the laboratory under tap water and divided into three sub-samples. One sub-sample was immediately frozen in liquid nitrogen and stored at −80 °C until its use for metabolomics analysis. The other one was stored at −20 °C and used for AM fungal colonization measurements and molecular analyses. The last was dried at 105 °C for 1 h, then at 75 °C for 48 h to determinate the dry weights and the inorganic ion concentrations. Soil around roots was also collected to determine the hyphal length density of AM fungi and the population density of *T. longibrachiatum* MF.

### 2.5. Determination of Colonization and Hyphal Length Density of AM Fungi

AM fungal colonization was assessed by staining roots with Trypan blue and scoring using the magnified intersection method. In total, three hundred random intersections were taken from each root sample as described by McGonigle et al. [31]. AM fungal hyphal length density in soil was measured using the gridline intersect method at 200× magnification followed by Miller et al. [32].

### 2.6. The Population of T. longibrachiatum MF

The population density of *T. longibrachiatum* was determined by plate count as described by Efthymiou et al. [33]. Briefly, 5 g soil samples collected from each pot were shaken in 45 mL of sterile Milli-Q water for 30 min at 120 rpm. Then, 100 µL aliquots were plated on Potato Dextrose Agar (PDA) with ampicillin (100 µg·mL^−1^). Plates were incubated at 30°C for five days, after which the number of colonies was assessed.

### 2.7. AM Fungal Community Structure Analysis

AM fungal community composition in maize roots was analyzed using PCR amplicon sequencing of the small subunit (SSU) rRNA gene. The DNA was extracted from 200–300 mg of root sample using a Fast Plant DNA Extraction Kit [29]. Glomeromycotina SSU rRNA gene sequences were amplified with the primers AML1F/AML2R and AMV4-5NF/AMDGR, which are designed to have high genus-level resolution for AM fungal communities [34]. The PCR sample was used for high-throughput sequencing with an Illumina MiSeq platform by Shanghai Majorbio Bio-pharm Technology Co., Ltd. (Shanghai, China) as described by Yang et al. [29].

Sequences read with ambiguous nucleotides or those that were lacking a complete barcode and primer were excluded. Chimeric sequences were identified and removed using UCHIME [35]. Operational taxonomic units (OTUs) with a 97% similarity cutoff were clustered using a searching platform (version 7.0; http://drive5.com/uparse/, accessed on 10 January 2021). For each OTU, the sequence numbers <5 were removed from analysis. The most abundant sequence from each OTU was selected as a representative sequence for that OTU. The taxonomy of representatives from each OTU was checked using the online MaarjAM database (http://www.maarjam.botany.ut.ee/, accessed on 10 January 2021, [36]) to determine whether these sequences belonged to Glomeromycota. Only sequences confirmed as AM fungi were included in the subsequent analysis. One representative sequence in each OTU clade was blasted against the public databases on the NCBI website to obtain three well-identified reference sequences. The representative sequences of our OTUs and the reference sequences were aligned to create a neighbor-joining (NJ) phylogenetic tree using MEGA software (version 5). The raw sequencing data were deposited to the NCBI sequence read archive (SRA) under the accession number PRJNA739072.

### 2.8. Profiling of Metabolites

The LC-MS system for metabolomics analysis is composed of Waters Acquity I-Class PLUS ultra-high performance liquid tandem Waters Xevo G2-XS QT high resolution mass spectrometer. The column that was used was purchased from Waters Acquity UPLC HSS T3 column (1.8 um 2.1 × 100 mm). Positive ion mode: mobile phase A: 0.1% formic acid aqueous solution; mobile phase B: 0.1% formic acid acetonitrile. Negative ion mode: mobile phase A: 0.1% formic acid aqueous solution; mobile phase B: 0.1% formic acid acetonitrile.

Waters Xevo G2-XS QTOF high resolution mass spectrometers can collect primary and secondary mass spectrometry data in MSe mode under the control of the acquisition software (MassLynx V4.2, Waters, Milford, MA, USA). In each data acquisition cycle, dual-channel data acquisition can be performed on both low collision energy and high collision energy at the same time. The low collision energy is 2 V, the high collision energy range is 10~40 V, and the scanning frequency is 0.2 s for a mass spectrum. The parameters of the ESI ion source are as follows: Capillary voltage: 2000 V (positive ion mode) or −1500 V (negative ion mode); cone voltage: 30 V; ion source temperature: 150 °C; desolvent gas temperature 500 °C; backflush gas flow rate: 50 L/h; Desolventizing gas flow rate: 800 L/h.

The raw data collected using MassLynx V4.2 were processed by Progenesis QI software for peak extraction, peak alignment, and other data processing operations, based on the Progenesis QI software online METLIN database and Biomark’s self-built library for identification, and at the same time, theoretical fragment identification and mass deviation were all determined to be within 100 ppm.

After normalizing the original peak area information with the total peak area, the follow-up analysis was performed. A principal component analysis and a Spearman correlation analysis were used to judge the repeatability of the samples within the group and the quality control samples. The identified compounds were searched for classification and pathway information in KEGG, HMDB, and lipidmaps databases. According to the grouping information, a *T* test was used to calculate the difference significance *p* value of each compound. The different metabolites of the KEGG pathway enrichment significance were calculated using hypergeometric distribution test. The metabolic compounds and pathway analysis was performed using BMK Cloud (www.biocloud.net). For the above experiments, roots were harvested per biological replicate, and six independent biological replicates were used.

### 2.9. Data Analysis

The statistical analyses were performed using R, version 3.5.2 (http://www.r-project.org/). The rarefaction curves of the AM fungal communities were calculated using the ‘vegan’ package in R [37]. The rarefaction curves suggested that a large proportion of the total AM fungal diversity colonizing roots under different treatments had been captured (Figure S2). Non-metric multidimensional scaling (NMDS) based on Bray-Curtis distances was used to show dissimilarities in the structure of the AM fungal communities and metabolic compounds (using the ‘metaMDS’ function in R). To determine the significance of the effects of the salinity levels, inoculants, and their interaction on the structure of the AM fungal communities and metabolic compounds, permutational multivariate analysis of variance was used (PERMANOVA; function: ‘adonis’ in R [38]). To determine whether particular AM fungal indicator OTUs made a greater contribution to the different salinity and inoculant treatments, the ‘multipatt’ function in the ‘indicspecies’ library was used [39]. OTUs with IndVal values ≥ 0.5 and *p* ≤ 0.05 were recorded as indicator species of differences among groups. The impact of salinity levels, inoculants, and their interactions on plant growth and AM fungal performance (in roots and soil) was analyzed by multivariate analysis of variance (MANOVA) followed by univariate analysis of variance (ANOVA) with the generalized linear model (GLM). Differences in plant biomass, shoot K^+^/Na^+^ ratio under the two salinity conditions (in Experiment 2) were assessed by one-way ANOVA, and significant differences among treatments were tested using Tukey’s honestly significant difference (HSD) test at a 95% confidence level. The significant differences of AM fungal performance between non-inoculated and MF inoculated treatments in experiment 1 and differences between Gm inoculated and Gm + MF inoculated treatments in experiment 2 were tested using a *t*-test at a 95% confidence level. These statistical analyses were performed using the SPSS software package version 21.0 (SPSS Inc., Chicago, IL, USA).

## 3. Results

### 3.1. Experiment 1

#### 3.1.1. *T. longibrachiatum* MF, AM Fungi and Maize Performance

At harvest, the population density of *T. longibrachiatum* MF was significantly higher in the MF inoculated treatments than the non-inoculated treatments at all salinity conditions (Table S2). Low numbers of MF colonies were detected in the treatments receiving no inoculum; however, these colonies differed from the MF strain’s phenotype and thus were considered as background colonies. The effects of salinity, inoculants, and their interactions with the CFU values of MF strains were all significant, and the CFU values of MF in soil decreased with increasing soil salinity (Table S2).

**Figure 1:**
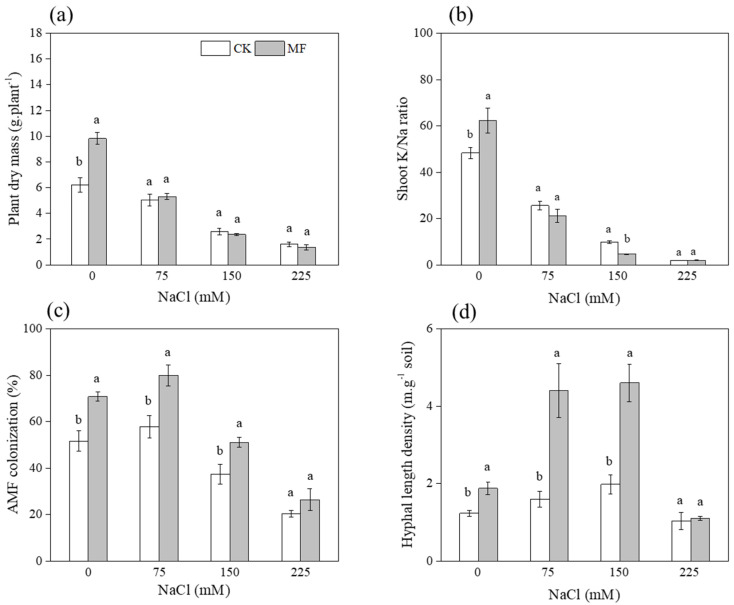
Effects of salinity levels (0, 75, 150, and 225 mM NaCl conditions) and inoculum types (CK and MF inoculated treatment) on plant dry mass (**a**), shoot K^+^/Na^+^ ratio (**b**), AM fungal colonization rate in root (**c**), and hyphal length density in soil (**d**), respectively. Values are mean ± SE (*n* = 3). Different lowercase letters indicate significant differences between CK and MF. Inoculated treatments were tested using a *t*-test at 95% confidence level.

Maize biomass was influenced by salinity, MF inoculant and their interactions (Table 1). MF inoculant improved maize biomass by 58.3% over that of non-inoculated maize with non-saline conditions (Figure 1a). Shoot K^+^/Na^+^ ratio was influenced by salinity and salinity × MF inoculant interaction (Table 1). MF inoculant increased shoot K^+^/Na^+^ ratio by 29.2% compared to that of non-inoculated maize but only at the non-saline condition (Figure 1b).

The AM fungal colonization rate was influenced by salinity and MF inoculant significantly (Table 1). The colonization rate of MF inoculated maize increased by 36.9%, 38.3%, and 37.0% over that of non-inoculated maize at 0, 75, and 150 mM NaCl conditions, respectively. (Figure 1c). Hyphal length density was influenced by salinity, MF inoculant, and their interactions (Table 1). The significant increase of hyphal length density in MF inoculated soil was found at 0, 75, and 150 mM NaCl conditions (Figure 1d).

#### 3.1.2. AM Fungal Structure in Maize Roots

**Figure 2:**
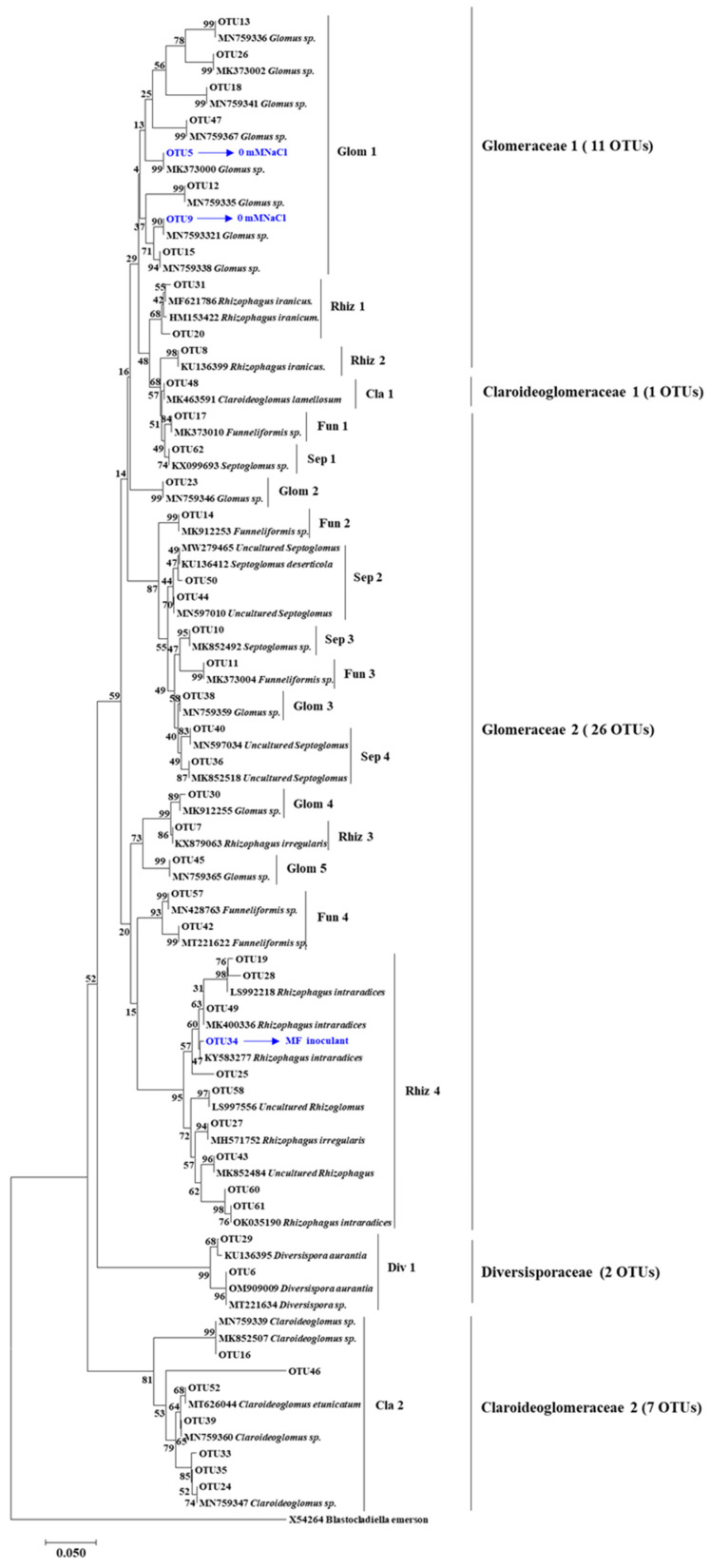
Neighbor-joining phylogram of OTUs showing the phylogenetic relationship of the AM fungal sequences obtained from maize roots. Numbers above branches denote bootstrap values from 1000 replications. Different salinity levels (0, 75, 150, and 225 mM NaCl conditions) and inoculum types (CK and MF). The indicator species are depicted using blue characters.

**Figure 3:**
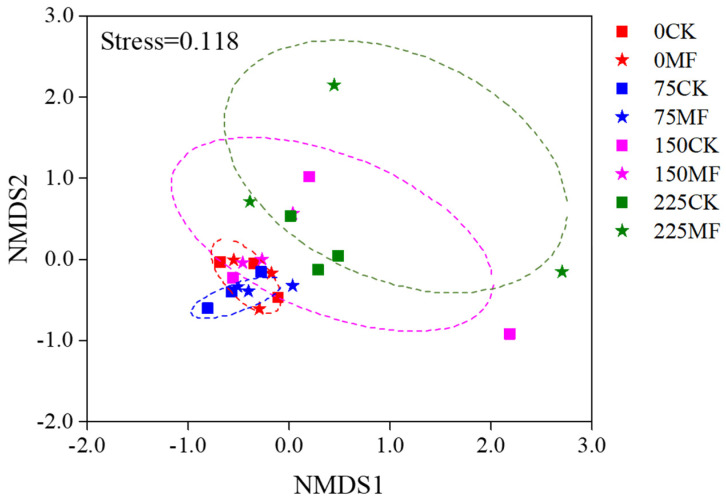
Non-metric multidimensional scaling (NMDS) plot of the AM fungal communities in maize roots under different salinity levels. Red symbols: 0 NaCl, blue symbols: 75 NaCl, purple symbols: 150 NaCl, and green symbols: 225 NaCl. Square: CK, Pentagram: MF inoculated treatment. Ellipses represent 95% confidence intervals around the centroid of all samples.

A total of 553,460 Illumina-generated valid sequences were retrieved from 24 root samples. Among these, 552,781 sequences were AM fungi (Table S3). Sequences were classified into 47 OTUs in three families based on sequence similarity (>97%): Glomeraceae contained 37 OTUs, Claroideoglomeraceae contained 8 OTUs, and Diversisporaceae contained 2 OTUs (Figure 2). At the OTU level, there were two indicator species (OTU5 and OTU9) for the 0 mM NaCl condition and only one indicator species (OTU34) for the MF inoculated treatment (Figure 2). The composition of AM fungal communities differed among the salinity levels, but the effects of MF inoculant or salinity × MF inoculant interaction were not significant (Figure 3). The AM fungal communities were differentiated between 0 and 225 mM NaCl conditions, significantly (Figure 3).

### 3.2. Experiment 2

#### 3.2.1. *T. longibrachiatum* MF, AM Fungi and Maize Performance

The population density of *T. longibrachiatum* MF was slightly lower at 0 mM NaCl conditions than those at 75 mM NaCl conditions in the MF and Gm + MF inoculated treatments (Table S4). The effects of salinity, Gm inoculants, and their interactions with the CFU values of MF strains were all not significant (Table S4).

**Figure 4:**
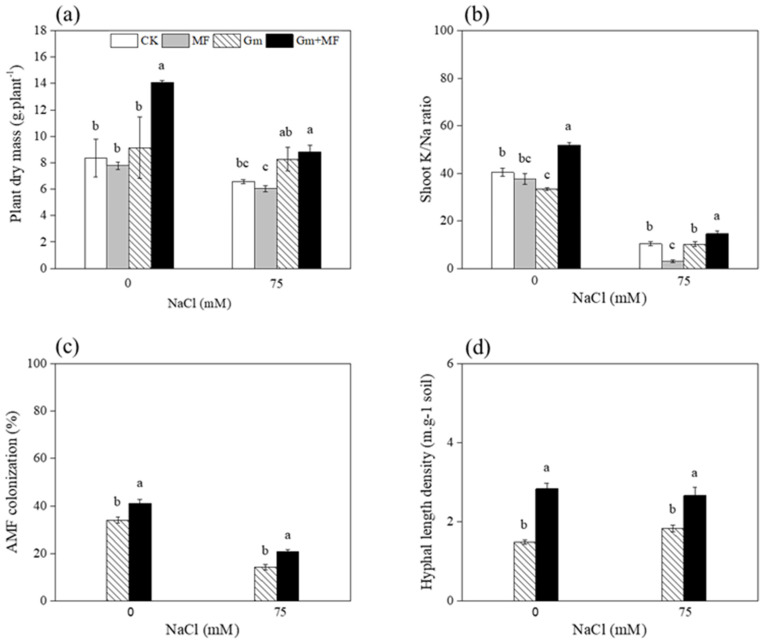
Effects of salinity levels (0 and 75 mM NaCl conditions) and inoculum types (CK, MF, Gm, and Gm + MF inoculated treatments) on plant dry mass (**a**), shoot K^+^/Na^+^ ratio (**b**), AM fungal colonization rate in roots (**c**), and hyphal length density in soil (**d**), respectively. Values are mean ± SE (*n* = 3). Different lowercase letters indicate significant differences of plant dry mass and shoot K^+^/Na^+^ ratio among treatments that were tested using Tukey’s honestly significant difference (HSD) test at a 95% confidence level. Significant differences of AM fungal performance between Gm and Gm + MF inoculated treatments were tested using a *t*-test at 95% confidence level.

Maize biomass was influenced by salinity and inoculants (Table 1). MF or Gm inoculant did not influence the maize growth significantly, however, Gm + MF inoculants significantly improved maize biomass by 68.6% and 34.5% over that of the non-inoculated maize at 0 and 75 mM NaCl conditions (Figure 4a). Shoot K^+^/Na^+^ ratio was influenced by salinity, inoculum, and salinity × inoculum interaction (Table 1). MF or Gm inoculant did not affect the shoot K^+^/Na^+^ ratio significantly, however, Gm + MF inoculants significantly increased shoot K^+^/Na^+^ ratio by 28.4% and 40.5% compared with that of non-inoculated maize at 0 and 75 mM NaCl conditions (Figure 4b).

The AM fungal colonization rate was influenced by salinity and inoculants significantly (Table 1). The colonization rate of Gm + MF inoculated maize increased by 20.7% and 44.8% over that of non-inoculated maize at 0 and 75 mM NaCl conditions. (Figure 4c). Hyphal length density was influenced by inoculants, and salinity levels did not affect the hyphal length density (Table 1). The hyphal length density of Gm + MF inoculated soil increased by 91.3% and 45.2% over those of non-inoculated at 0 and 75 mM NaCl conditions, respectively (Figure 4d).

#### 3.2.2. Metabolic Profiles in Maize Roots

**Figure 5:**
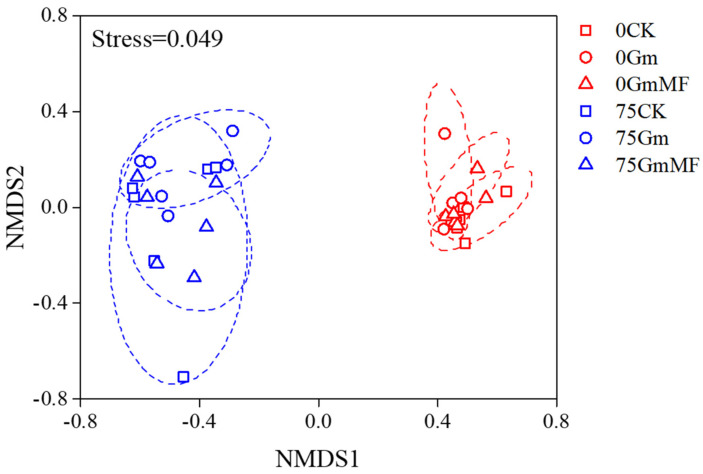
Overview of the metabolomic reprogramming in maize plant roots under CK, Gm treatment, and Gm + MF treatment at 0 and 75 mM NaCl conditions. Red symbols: 0 NaCl and blue symbols: 75 NaCl. Square: CK, circle: Gm and triangle: Gm + MF. Ellipses represent 95% confidence intervals around the centroid of all samples.

The LC-MS analysis identified a total of 189 metabolites in maize roots in the CK, Gm, and Gm + MF treatments at 0 and 75 mM NaCl conditions. Table S1 lists the metabolites detected in all roots. The salinity had a strong impact on metabolites, while the metabolites remained unaffected either by Gm or Gm + MF (Figure 5). However, metabolite profile analysis showed that Gm and Gm + MF treatment have important effects on root metabolites at both salinity conditions. (Table 2). The metabolites with higher accumulation in Gm inoculated treatment were generally different from those in Gm + MF treatment, and Gm + MF treatment up-regulated five metabolites and down-regulated two metabolites (Table 2). Seven compounds displaying the strongest changes in accumulation confirmed that Gm + MF treatment has an important impact on root metabolites at 0 and 75 mM NaCl conditions.

**Figure 6:**
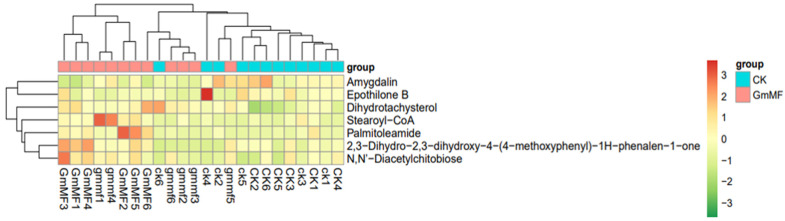
The heat map of differential metabolites accumulated in maize plant roots under un-inoculated and Gm + MF inoculated treatments at 0 and 75 mM NaCl conditions. CK, un-inoculated treatment, GmMF, Gm + MF inoculated treatment. Lower case letters (ck and gmmf) stand for 0 mM NaCl treatment, uppercase letters (CK and GmMF) stand for 75 mM NaCl treatment. *n* = 6.

Plants accumulated different compounds in response to different inoculation treatments. Among the different metabolites, we focused on two different types of clusters (Figure 6). On one hand, a group of compounds markedly up-regulated in response to Gm + MF treatment, including *N*,*N*′-Diacetylchitobiose, 2,3-Dihydro-2,3-dihydroxy-4- (4-methoxyphenyl), Palmitoleamide, Stearoyl-CoA, and Dihydrotachysterol. On the other hand, those common metabolites, in the same fashion as Amygdalin and Epothilone B, down-regulated in Gm + MF treatment. Considering that Gm + MF treatment promoted maize growth and shoot K^+^/Na^+^ ratio at 0 and 75 mM NaCl conditions, some of the metabolites may contribute to plant performance and salt tolerance. Among the metabolites, the *N*,*N*′-Diacetylchitobiose and Stearoyl-CoA were mainly related to the Amino sugar and nucleotide sugar metabolism as well as fatty acid metabolism in Gm + MF inoculated plants (Table S5). The Amygdalin was related to the Cyanoamino acid metabolism in Gm and Gm + MF inoculated plants (Table S5).

## 4. Discussion

The results of our experiments showed the interaction between AM fungi and *T. longibrachiatum* and their effects on the maize plant growth and plants’ metabolic profiles in saline soil. Several reports have shown a positive effect of dual inoculation with AM fungi and PGPM on plant growth and stress tolerance, such as AM fungi with *Pseudomonas mendocina* on lettuce [40], AM fungi with *Pseudomonas fluorescens* on common beans [41], AM fungi with *Azospirillum* on rice [42], and AM fungi with *Enterobacter radicincitans* on fava beans [43], but little is known about the interactions with *T. longibrachiatum* as well as the underlying mechanisms. In this study, we observed a significant growth benefit of the synergistic association of maize with AM fungi and *T. longibrachiatum* under salt stress.

### 4.1. T. longibrachiatum MF Improved AM Fungi Growth

First, we hypothesized that *T. longibrachiatum* MF inoculant improves AM fungi growth under salt stress. Our results clearly showed that the better root colonization and hyphal length density in MF inoculated treatment at 0, 75, and 150 mM NaCl conditions, while the lack of response to MF inoculant at 225 mM NaCl conditions, suggested that extreme saline is a limiting factor for microbe growth and interactions. Garg and Chandel [44] have found that a very high salinity decreased hyphal growth, which might have been due to the inhibition of spore germination. Despite the detrimental effects of salinity on AM fungal association that have been observed [15], our results indicated that dual inoculation mitigated these effects.

The former study demonstrated that soil inoculation with *Trichoderma* was shown to increase the overall abundance of bacteria and affected the bacterial community structure in saline soil [9]. In this study, we found that the *T. longibrachiatum* inoculum significantly increased AM fungal biomass in roots and soil not only under non-stressed conditions but also under saline stress. *T. longibrachiatum* alleviated the harmful effects of salt stress in AM fungal colonies in roots, which suggested an additional value to the use of *T. longibrachiatum* associated with AM fungi in saline soil. PGPM are known to assist rhizospheric fungi in colonizing the roots of their host plants through the production of metabolites that increase cell permeability and stimulate hyphal growth by enhancing root exudation rates [45]. This synergy might have led to higher colonization rates by the AM fungi, improving the plants’ ability to manage salt stress [46], which might have contributed to the outstanding performance of the dual-inoculated treatment.

The effect of *T. longibrachiatum* on the structure of AM fungal communities in saline soil has sparsely been investigated. Our data indicated the composition of AM fungal communities differed substantially between 0 and 225 mM NaCl conditions, however, *T. longibrachiatum* MF had few impacts on AM fungal community composition. Our results supported the former evidence highlighting the importance of abiotic rather than biotic factors on the composition of the AM fungal communities [47]. Indicator species analyses identified that OTU_5 (*Glomus* sp.) and OTU_9 (*Glomus* sp.) were characteristic for 0 mM NaCl conditions. OTU_5 is a widespread taxon found in Estonian boreal forest and OTU_9 is a widespread taxon found in the grassland habitats [48]. It is possible that these species were uncompetitive in stressful environments. OTU_34 (*Rhizophagus* sp.) is characteristic for MF inoculated treatment, which has been found in the long-term monocultures agro-ecosystems [49].

### 4.2. The Dual Inoculation Enhanced Plant Growth

Second, we hypothesized that dual inoculation outperforms single inoculation under salt stress, and our results partly support this hypothesis. As for the plant growth, dual inoculation may be more effective in elevated maize plant performance than either of the single inoculations under non-saline condition but not under saline-stressed conditions. However, dual inoculation of *Glomus* sp. And *T. longibrachiatum* MF significantly increased shoot K^+^/Na^+^ ratio in plant tissues compared with single inoculated plants under salt stress.

In a former study, Fu et al. [9] found that *Trichoderma* spp. increased the maize yield by 12.4% and Pang et al. [50] discovered that *Trichoderma*-treated soil increased plant biomass by 20%. We observed that Gm + MF treatment significantly improved maize plant biomass by 68.6% and 34.5% over that of non-inoculated maize at 0 and 75 mM NaCl conditions. Dual inoculation with *Glomus* sp. And *T. longibrachiatum* MF was beneficial for the enhancement plant growth even in saline conditions. Similar results were observed by Helena et al. [51] combined inoculation of two PGPB strains and one AM fungal isolate increased maize biomass by 35% at 5 g NaCl kg^−1^ soil. Dual inoculation of AM fungi and PGPB enhanced alfalfa yield in salt-affected soils [52]. Osorio et al. [53] also found a greater contribution to plant nutrition by dual inoculation with a phosphate-solubilizing fungus and an AM fungus under salt stress. As beneficial soil microorganisms, the additive effects of AM fungi and PGPM could be an attractive ecological solution for enhancing plant growth when the plants are exposed to higher stress.

The extent of plant sensitivity to salinity depends mainly on Na^+^ uptake, accumulation, and its root-shoot distribution [54]. Chang et al. [55] has found that AM fungi modulates antioxidant response and ion distribution under salt stress. The presence of AM symbiosis mitigated the salinity-induced increase in shoot Na^+^ concentration [56]. In our study, dual inoculation of *Glomus* sp. and *T. longibrachiatum* MF enhanced shoot K^+^/Na^+^ ratio by 40.5% under salt stress, which may be a collective result of the many positive changes induced by AM fungi and PGPM. In salt-stressed *Acacia gerrardii*, dual-inoculated plants with AM fungi and PGPM reduced the Na^+^ concentration in plant tissues, thereby protecting salt-stressed plants from ionic and osmotic stress-induced changes [57]. When dual-inoculated with *Glomus* sp. and *T. longibrachiatum* MF, Na^+^ may either be stored in intraradical AM fungal hyphae or compartmentalized in the root cell vacuoles [58], thus preventing their translocation to the plant shoots. The dual inoculation of *Glomus* sp. and *T. longibrachiatum* MF presents a promising avenue to the control of Na^+^ accumulation and therefore the capacity to augment salt tolerance in maize. In the future, MF may be a potential candidate for use in new bio-fertilizers for maintaining appropriate Na^+^ concentration in maize plant in environmentally stressed soils.

### 4.3. The Dual Inoculation Modulated Root Metabolome

It has previously been found that dual inoculation of different PGPM can enhance plant tolerance to salt stress [57], however, few studies have investigated their impacts on the host stress-induced reorganization of the metabolome in order to identify key metabolites involved in dual inoculation related protection against salt stress. In this study, through untargeted metabolic analysis, the major metabolic pathways related to plant tolerance to salt stress were deduced. Our results clearly indicated that Gm + MF treatment up-regulated five metabolites and down-regulated two metabolites, which may help the plant to maintain an elevated carbohydrate metabolism and fatty acid metabolism even in saline soil, which supported our third hypothesis.

The metabolic profiles of maize roots under Gm + MF treatments were compared with those under non-inoculated treatment both at 0 and 75 mM NaCl conditions. Seven compounds’ accumulation changed significantly in dual inoculation, and this phenomenon can be explained by the different strategies of plants in coping with inoculant treatments [51]. The higher accumulation of *N*,*N*′-Diacetylchitobiose and Stearoyl-CoA were observed regardless of salt stress under Gm + MF treatment, which probably linked to plant performance or tolerance demands. Stearoyl-coA was closely related to the synthesis of plant unsaturated fatty acids, such as phospholipids, triglycerides, wax esters, and cholesterol esters [59]. All the plants in Gm + MF treatment showed a higher accumulation of Stearoyl-coA, which is important in the enhanced biosynthesis of unsaturated fatty acids. The accumulation of N, N’-Diacetylchitobiose is closely related to the amino sugar and nucleotide sugar metabolism pathway. These observations suggest that Gm + MF treatment could help the plant to maintain an elevated carbohydrate metabolism and fatty acid metabolism, which may lead to an increasing of plant resistance under salt stress. In fact, plants must integrate metabolites adaptive to environmental stress, such as the accumulation of solasodine in response to salt stress [60], and the accumulation of hytohormone jasmonic acid when AM fungi inoculant is applied to saline soil [61]. The root metabolome of salt-affected plants can be buffered with a biostimulant, which can contribute to an overall stress reduction of plants.

## 5. Conclusions

In summary, two experiments in this study allowed us to find the changes of the plant in response to dual inoculation in saline soil. Our results suggest that AM fungi and *T. longibrachiatum* MF living in the rhizosphere of maize were coordinately involved in the plant’s adaptation to salt stress tolerance. Dual inoculation of plants with *Glomus sp*. and *T. longibrachiatum* MF increased plant performance through the alteration of metabolic profiles under salt stress. The combination of the most suitable PGPMs may lead to the mitigation of the inhibitive impact of salt stress on AM fungal association as well as the improvement of the optimum tolerance of maize growth in agro-ecosystems.

## Figures and Tables

**Table 1:** Summary of statistical analysis testing the differences between salinity levels, inoculum types, and their interactions with plant dry mass, shoot K^+^/Na^+^ ratio, AMF colonization rate, and hyphal length density (HLD) (experiment 1 and experiment 2). Three replicates per treatment combination are included. *F*-values are accompanied by indicators of statistical significance: *, *p* < 0.05; **, *p* < 0.01; ***, *p* < 0.001, and ns, non-significant.

Parameters	Salinity (*S*)	Inoculants (*I*)	Interaction *S×I*
*Experiment 1*			
Plant dry mass	146.43 ***	12.68 **	14.63 ***
Shoot K^+^/Na^+^ ratio	199.86 ***	0.42 ns	6.97 **
AMF colonization rate	57.06 ***	32.60 ***	1.74 ns
HLD	20.53 ***	41.30 ***	8.34 **
*Experiment 2*			
Plant dry mass	10.85 **	7.60 **	1.75 ns
Shoot K^+^/Na^+^ ratio	1282.87 ***	43.47 ***	12.61 ***
AMF colonization rate	233.02 ***	26.52 **	0.54 ns
HLD	0.41 ns	63.82 ***	3.69 ns

**Table 2:** Effects of single and dual-inoculants on the metabolite profiles in maize plant roots.

ID	Metabolite Name	CK vs. Gm	CK vs. Gm + MF	Gm vs. Gm + MF
meta_804	Amygdalin	down	down	
meta_445	Caproic acid			down
meta_430	6,10,14-Trimethyl-5,9,13-pentadecatrien-2-one	up		
meta_291	Epothilone B		down	
meta_544	Tylosin	up		
meta_188	Pro Met Met Thr	down		
meta_196	Chamazulene	down		
meta_701	2,3-Dihydro-2,3-dihydroxy-4- (4-methoxyphenyl) -1H-phenalen-1-one		up	
meta_696	Stearoyl-CoA		up	
meta_753	Dihydrotachysterol		up	
meta_789	*N*,*N*′-Diacetylchitobiose		up	
meta_627	3-Hydroxydodecanoic acid	up		
meta_432	Palmitoleamide		up	
meta_239	C16 Sphinganine	up		down
meta_747	*N*-Acetyl-a-neuraminic acid			up

## Data Availability

The representative sequences from each of the AM fungal OTUs obtained in this study have been submitted to GenBank, with the accession numbers MN759327-MN759373 (https://www.ncbi.nlm.nih.gov/search/, accessed on 10 January 2021). The raw sequencing data are deposited to the NCBI sequence read archive (SRA) under the accession number PRJNA739072 (https://www.ncbi.nlm.nih.gov/sra/PRJNA739072, accessed on 10 January 2021).

## Supplementary Materials

The following supporting information can be downloaded at: https://www.mdpi.com/article/10.3390/microorganisms10051042/s1, Figure S1. The growth of *Trichoderma longibrachiatum* MF on the Potato Dextrose Agar (PDA) plates with different salinity levels. Figure S2. Rarefaction curves for AM fungal phylotypes detected in roots (*n* = 24) at four levels salinity (0, 75, 150, and 225 mM NaCl) and inoculum types (CK and MF inoculated treatment). Table S1. 189 all metablics. Table S2. *T. longibrachiatum* population density in different salinity levels and inoculants. Values are mean ± SE (*n* = 3). Different asterisk indicate a significant effect of salinity, inoculant and their interaction according to Duncan’s multiple range test following significant two-way ANOVA, * *p* < 0.05, ** *p* < 0.01, *** *p* < 0.001 and ns, not significant. Different lowercase letters indicate significant differences between inoculant within each salinity level according to Duncan’s multiple range test following significant one-way ANOVA (*p* < 0.05). Table S3. Operational taxonomic units (OTU) of AM fungi detected in roots (*n* = 24) at four levels salinity (0, 75, 150, and 225 mM NaCl) and inoculum types (CK and MF inoculated treatment). Table S4. *T. longibrachiatum* population density in different salinity levels and inoculum treatments. Values are mean ± SE (*n* = 3). Different asterisk indicate a significant effect of salinity, inoculum and their interaction according to Duncan’s multiple range test following significant two-way ANOVA, * *p* < 0.05, ** *p* < 0.01, *** *p* < 0.001 and ns, not significant. Different lowercase letters indicate significant differences among inoculum within each salinity level according to Duncan’s multiple range test following significant one-way ANOVA (*p* < 0.05). Table S5. Effects of single inoculant and bio-inoculants on the metabolic pathways.

## References

[1] AbdElgawad H., Zinta G., Hegab M.M., Pandey R., Asard H., Abuelsoud W. (2016). High salinity induces different oxidative stress and antioxidant responses in maize seedlings organs. Front. Plant Sci..

[2] Hidri R., Barea J.M., Metoui-Ben M.O., Abdelly C., Azcon R. (2016). Impact of microbial inoculation on biomass accumulation by Sulla carnosa provenances, and in regulating nutrition, physiological and antioxidant activities of this species under non-saline and saline conditions. J. Plant Physiol..

[3] Vimal S.R., Singh J.S., Arora N.K., Singh S. (2017). Soil-plant-microbe interactions in stressed agriculture management: A review. Pedosphere.

[4] Busby P.E., Soman C., Wagner M.R., Friesen M.L., Kremer J., Bennett A. (2017). Research priorities for harnessing plant microbiomes in sustainable agriculture. PLoS Biol..

[5] Toju H., Peay K.G., Yamamichi M., Narisawa K., Hiruma K., Naito K., Fukuda S., Ushio M., Nakaoka S., Onoda Y. (2018). Core microbiomes for sustainable agroecosystems. Nat. Plants.

[6] Hidri R., Mahmoud M.B., Debez A., Abdelly C., Azcon R. (2019). Modulation of c:n:p stoichiometry is involved in the effectiveness of a PGPR and am fungus in increasing salt stress tolerance of sulla carnosa tunisian provenances. App. Soil Ecol..

[7] Ghaffari M.R., Ghabooli M., Khatabi B., Hajirezaei M.R., Schweizer P., Salekdeh G.H. (2016). Metabolic and transcriptional response of central metabolism affected by root endophytic fungus Piriformospora indica under salinity in barley. Plant Mol. Biol..

[8] Gul Jan F., Hamayun M., Hussain A., Jan G., Iqbal A., Khan A., Lee I.-J. (2019). An endophytic isolate of the fungus yarrowia lipolytica produces metabolites that ameliorate the negative impact of salt stress on the physiology of maize. BMC Microbial..

[9] Fu J., Xiao Y., Wang Y.F., Liu Z.H., Yang K.J. (2019). Trichoderma affects the physiochemical characteristics and bacterial community composition of saline-alkaline maize rhizosphere soils in the cold-region of Heilongjiang province. Plant Soil.

[10] Harman G.E., Howell C.R., Viterbo A., Chet I., Lorito M. (2004). Trichoderma species-opportunistic, avirulent plant symbionts. Nat. Rev. Microbial..

[11] Bae H., Roberts D.P., Lim H.S., Strem M.D., Park S.C., Ryu C.M. (2011). Endophytic trichoderma isolates from tropical environments delay disease onset and induce resistance against phytophthora capsici in hot pepper using multiple mechanisms. Mol. Plant Microbial. Interact..

[12] Wazny R., Rozpadek P., Jedrzejczyk R.J., Sliwa M., Stojakowska A., Anielska T., Turnau K. (2018). Does co-inoculation of *lactuca serriola* with endophytic and arbuscular mycorrhizal fungi improve plant growth in a polluted environment?. Mycorrhiza.

[13] Nadeem S.M., Ahmadm M., Zahir Z.A., Javaid A., Ashraf M. (2014). The role of mycorrhizae and plant growth promoting rhizobacteria (PGPR) in improving crop productivity under stressful environments. Biotech. Adv..

[14] Egamberdieva D., Li L., Lindström K., Räsänen L. (2016). A synergistic interaction between salt tolerant Pseudomonas and Mezorhizobium strains improves growth and symbiotic performance ofliquorice (*Glycyrrhiza uralensis* Fish.) under salt stress. Apply Microbial. Biotechnol..

[15] Ruiz-Lozano J.M., Porcel R., Azcon C., Aroca R. (2012). Regulation by arbuscular mycorrhizae of the integrated physiological response to salinity in plants: New challenges in physiological and molecular studies. J. Exp. Bot..

[16] Astrit B., Glenda S., Boris R. (2015). AMF inoculation enhances growth and improves the nutrient uptake rates of transplanted, salt-stressed tomato seedlings. Sustainability.

[17] Porcel R., Aroca R., Ruiz-Lozano J.M. (2012). Salinity stress alleviation using arbuscular mycorrhizal fungi. A review. Agron. Sustain. Dev..

[18] Marulanda-Aguirre A., Azcon R., Ruiz-Lozano J.M., Aroca R. (2008). Differential effects of a Bacillus megaterium strain on Lactucasativa plant growth depending on the origin of the arbuscular mycorrhizal fungus coinoculated: Physiologic and biochemical traits. J. Plant Growth Regul..

[19] Toro M., Azcón R., Barea J.M. (1997). Improvement of arbuscular mycorrhiza development by inoculation of soil with phosphate-solubilizing rhizobacteria to improve rock phosphate bioavailability (32P) and nutrient cycling. Appl. Environ. Microbial..

[20] Lagos C., Larsen J., Correa E.S., Almonacid L., Héctor H., Fuentes A., Arriagada C. (2018). Dual inoculation with mycorrhizal and saprotrophic fungi suppress the maize growth and development under phenanthrene exposure. J. Soil Sci. Plant Nutr..

[21] Chandanie W.A., Kubota M., Hyakumachi M. (2009). Interactions between the arbuscular mycorrhizal fungus *Glomus mosseae* and plant growth promoting fungi and their significance for enhancing plant growth and suppressing damping-off of cucumber (*Cucumis sativus* L.). Appl. Soil Ecol..

[22] Singh S., Kapoor K.K. (1999). Inoculation with phosphate-solubilizing microorganisms and a vesicular-arbuscular mycorrhizal fungus improves dry matter yield and nutrient uptake by wheat grown in a sandy soil. Biol. Fertil. Soil.

[23] Osorio N.W., Habte M. (2015). Effect of a phosphate-solubilizing fungus and an arbuscular mycorrhizal fungus on leucaena seedlings in tropical soils with contrasting phosphate sorption capacity. Plant Soil.

[24] Deinlein U., Stephan A.B., Horie T., Luo W., Xu G., Schroeder J.I. (2014). Plant salt tolerance mechanisms. Trends Plant Sci..

[25] Rivero J., Álvarez D., Flors V., Azcon-Aguilar C. (2018). Root metabolic plasticity underlies functional diversity in mycorrhiza-enhanced stress tolerance in tomato. New Phytol..

[26] Rodziewicz P., Swarcewicz B., Chmielewska K., Wojakowska A., Stobiecki M. (2014). Influence of abiotic stresses on plant proteome and metabolome changes. Acta Physiol. Plant..

[27] Yang R., Wang J.J., Xu S., Zhao W., Liu H.Y., Li Q.Q., Huang Z.Y. (2018). Screening, identification and salt-tolerant characteristics of phosphate-solubilizing fungi. Microbiol. China.

[28] Yuan S.F., Li M.Y., Fang Z.Y., Liu Y., Shi W., Pan B., Wu K., Shi J.X., Shen B., Shen Q.R. (2016). Biological control of tobacco bacterial wilt using Trichoderma harzianum amended bioorganic fertilizer and the arbuscular mycorrhizal fungi *Glomus Mosseae*. Biol. Control.

[29] Yang R., Qin Z.F., Wang J.J., Xu S., Zhao W., Zhang X.X., Huang Z.Y. (2020). Salinity changes root occupancy by arbuscular mycorrhizal fungal species. Pedobiol. J. Soil Biol..

[30] Utobo E.B., Ogbodo E.N., Nwogbaga A.C. (2011). Techniques for extraction and quantification of arbuscular rmycorrhizal fungi. Libyan Agric. Res. Center Int..

[31] McGonigle T.P., Miller M.H., Evans D.G., Fairchild G.L., Swan J.A. (1990). A new method which gives an objective measure of colonization of roots by vesicular-arbuscular mycorrhizal fungi. New Phytol..

[32] Miller R.M., Reinhardt D.R., Jastrow J.D. (1995). External hyphal production of vesicular arbuscular mycorrhizal fungi in pasture and tallgrass prairie communities. Oecologia.

[33] Efthymiou A.K., Jensen B., Jakobsen I. (2018). The roles of mycorrhiza and penicillium inoculants in phosphorus uptake by biochar-amended wheat. Soil Biol. Biochem..

[34] Lumini E., Orgiazzi A., Borriello R., Bonfante P., Bianciotto V. (2010). Disclosing arbuscular mycorrhizal fungal biodiversity in soil through a land-use gradient using a pyrosequencing approach. Environ. Microbial..

[35] Edgar R.C., Haas B.J., Clemente J.C. (2011). UCHIME improves sensitivity and speed of chimera detection. Bioinformatics.

[36] Öpik M., Vanatoa A., Vanatoa E., Moora M., Davison J., Kalwij J.M., Reier Ü., Zobel M. (2010). The online database MaarjAM reveals global and ecosystemic distribution patterns in arbuscular mycorrhizal fungi (Glomeromycota). New Phytol..

[37] Kembel S.W., Cowan P.D., Helmus M.R. (2010). Picante: R tools for integrating phylogenies and ecology. Bioinformatics.

[38] Anderson M.J. (2001). A new method for non-parametric multivariate analysis of variance. Austral Ecol..

[39] de Caceres M., Legendre P., Moretti M. (2010). Improving indicator species analysis by combining groups of sites. Oikos.

[40] Kohler J., Hernandez J.A., Caravaca F., Roldan A. (2009). Induction of antioxidant enzymes is involved in the greater effectiveness of a PGPR versus AM fungi with respect to increasing the tolerance of lettuce to severe salt stress. Environ. Exp. Bot..

[41] Kanchan Singh N. (2011). Organic amendments to soil inoculated arbuscular mycorrhizal fungi and Pseudomonas fluorescens treatments reduce the development of root-rot disease and enhance the yield of *Phaseolus vulgaris* L.. Eur. J. Soil Biol..

[42] Ruíz-Sánchez M., Armada E., Munoz Y., de Salamone I.E.G., Aroca R., Ruíz-Lozano J.M., Azcón R. (2011). Azospirillum and arbuscular mycorrhizal colonization enhance rice growth and physiological traits under wellwatered and drought conditions. J. Plant Physiol..

[43] Almethyeb M., Ruppel S., Paulsen H.M., Vassilev N., Eichler-Löbermann B. (2013). Single and combined applications of arbuscular mycorrhizal fungi and *Enterobacter radicincitans* affect nutrient uptake of faba bean and soil biological characteristics. Agric. Forest Res..

[44] Garg N., Chandel S. (2015). Role of arbuscular mycorrhiza in arresting reactive oxygen species (ROS) and strengthening antioxidant defense in *Cajanus cajan* (L.) Millsp. Nodules under salinity (NaCl) and cadmium (Cd) stress. Plant Growth Regul..

[45] Jeffries P., Gianinazzi S., Perotto S., Turnau K., Barea J.M. (2003). The contribution of arbuscular mycorrhizal fungi in sustainable maintenance of plant health and soil fertility. Biol. Fertil. Soi..

[46] Krishnamoorthy R., Kim K., Subramanian P., Senthilkumar M., Anandham R., Sa T. (2016). Arbuscular mycorrhizal fungi and associated bacteria isolated from saltaffected soil enhances the tolerance of maize to salinity in coastal reclamation soil. Agric. Ecosyst. Environ..

[47] Van Geel M., Jacquemyn H., Plue J., Saar L., Ceulemans T. (2017). Abiotic rather than biotic filtering shapes the arbuscular mycorrhizal fungal communities of European seminatural grasslands. New Phytol..

[48] ÖPik M., Moora M., Liira J., Kõljalg U., Zobel M., Seb R. (2003). Divergent arbuscular mycorrhizal fungal communities colonize roots of *Pulsatilla* spp. in boreal scots pine forest and grassland soils. New Phytol..

[49] Yamato M., Ikeda S., Iwase K. (2008). Community of arbuscular mycorrhizal fungi in a coastal vegetation on okinawa island and effect of the isolated fungi on growth of sorghum under salt-treated conditions. Mycorrhiza.

[50] Pang G., Cai F., Li R., Zhao Z., Li R., Gu X., Shen Q.R., Chen W. (2017). Trichoderma-enriched organic fertilizer can mitigate microbiome degeneration of monocropped soil to maintain better plant growth. Plant Soil.

[51] Helena M., Sofia I.A.P., Alberto V., Paula M.L., Castro A.P.G.C.M. (2020). Synergistic effects of arbuscular mycorrhizal fungi and plant growth-promoting bacteria benefit maize growth under increasing soil salinity. J. Environ. Manag..

[52] Ashrafi E., Zahedi M., Razmjoo J. (2014). Co-inoculations of arbuscular mycorrhizal fungi and rhizobia under salinity in alfalfa. Soil Sci. Plant Nutr..

[53] Osorio N.W., Habte M. (2013). Synergistic effect of a phosphate-solubilizing fungus and an arbuscular mycorrhizal fungus on leucaena seedlings in an oxisol fertilized with rock phosphate. Botany.

[54] Evelin H., Giri B., Kapoor R. (2012). Contribution of Glomus intraradices inoculation to nutrient acquisition and mitigation of ionic imbalance in NaCl-stressed Trigonella foenum-graecum. Mycorrhiza.

[55] Chang W., Sui X., Fan X.X., Jia T.T., Song F.Q. (2018). Arbuscular mycorrhizal symbiosis modulates antioxidant response and ion distribution in salt-stressed *Elaeagnus angustifolia* seedlings. Front. Microbial..

[56] Amanifar S., Toghranegar Z. (2020). The efficiency of arbuscular mycorrhiza for improving tolerance of *valeriana officinalis* L. and enhancing valerenic acid accumulation under salinity stress. Ind. Crops Prod..

[57] Abeer H., Elsayed F.A., Abdulaziz A.A., Asma A.A., Stephan W., Dilfuza E. (2016). The Interaction between Arbuscular Mycorrhizal Fungi and Endophytic Bacteria Enhances Plant Growth of *Acacia gerrardii* under Salt Stress. Front. Microbial..

[58] Qiu Y.J., Zhang N.L., Zhang L.L., Zhang X.L., Wu A.P., Huang J.Y., Yu S.Q., Wang Y.H. (2020). Mediation of arbuscular mycorrhizal fungi on growth and biochemical parameters of ligustrum vicaryi in response to salinity. Physiol. Mol. Plant Pathol..

[59] Ntambi J.M., Miyazaki M. (2004). Regulation of stearoyl-CoA desaturases and role in metabolism. Prog. Lipid Res..

[60] Shilpha J., Satish L., Kavikkuil M., Joe Virgin Largia M., Ramesh M. (2015). Methyl jasmonate elicits the solasodine production and anti-oxidant activity in hairy root cultures of *Solanum trilobatum* L.. Ind. Crops Prod..

[61] Riemann M., Dhakarey R., Hazman M., Miro B., Kohli A., Nick P. (2015). Exploring jasmonates in the hormonal network ofdrought and salinity responses. Front. Plant Sci..

