# Supplementary material for: The Interactions between Arbuscular Mycorrhizal Fungi and Trichoderma longibrachiatum Enhance Maize Growth and Modulate Root Metabolome under Increasing Soil Salinity
Source: Microorganisms. 2022 May 17;10(5):1042. doi: 10.3390/microorganisms10051042 (PMC9142908; doi:10.3390/microorganisms10051042)
Supplement: Supplementary file 1 [file microorganisms-10-01042-s001.zip › Figures S1 and S2.pdf]

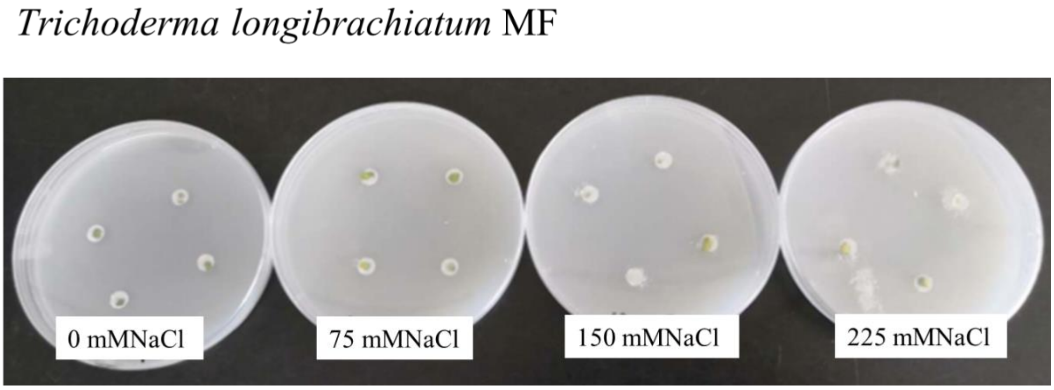

Figure S1. The growth of *Trichoderma longibrachiatum* MF on the Potato Dextrose Agar (PDA) plates with different salinity levels.

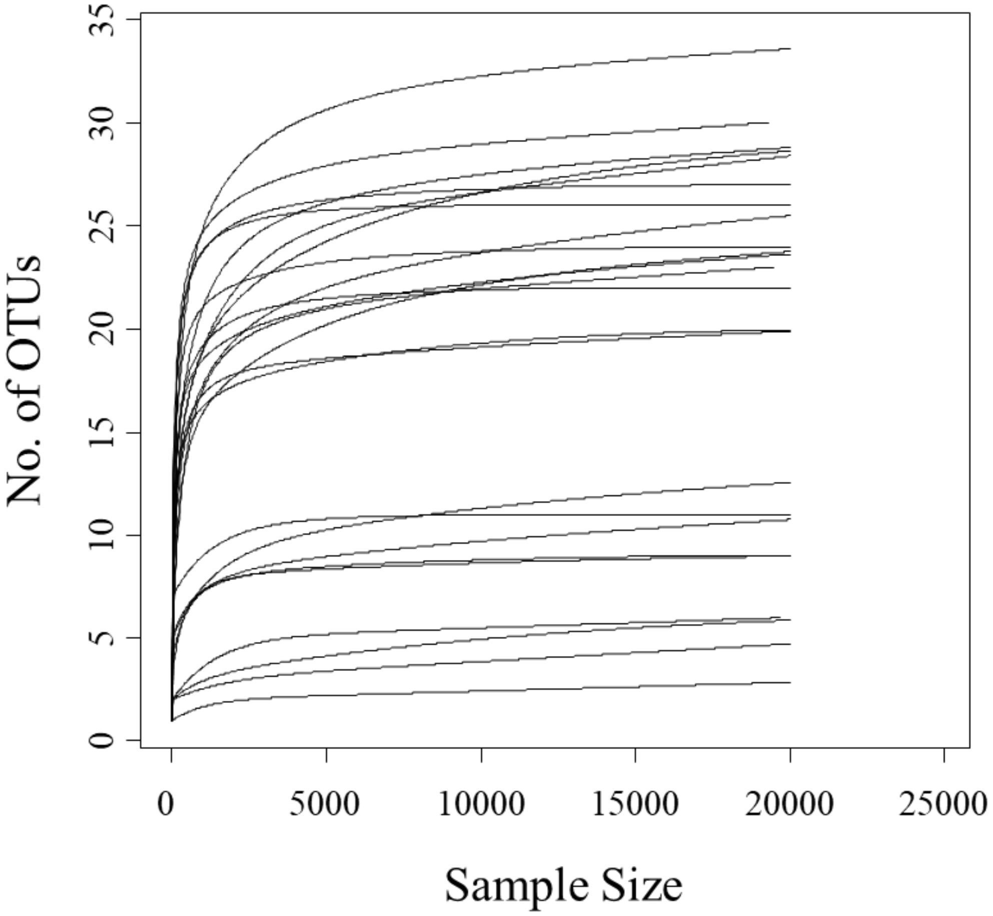

Figure S2. Rarefaction curves for AM fungal phylotypes detected in roots (n = 24) at four levels salinity (0, 75, 150, and 225 mM NaCl) and inoculum types (CK and MF inoculated treatment).
