# Supplementary material for: The Interactions between Arbuscular Mycorrhizal Fungi and Trichoderma longibrachiatum Enhance Maize Growth and Modulate Root Metabolome under Increasing Soil Salinity
Source: Microorganisms. 2022 May 17;10(5):1042. doi: 10.3390/microorganisms10051042 (PMC9142908; doi:10.3390/microorganisms10051042)
Supplement: Supplementary file 1 [file microorganisms-10-01042-s001.zip › Tables S2-S5.pdf]

Table S2. *T. longibrachiatum* population density in different salinity levels and inoculants. Values are mean ± SE (n=3). Different asterisk indicate a significant effect of salinity, inoculant and their interaction according to Duncan's multiple range test following significant two-way ANOVA, * $P < 0.05$, ** $P < 0.01$, *** $P < 0.001$ and ns, not significant. Different lowercase letters indicate significant differences between inoculant within each salinity level according to Duncan's multiple range test following significant one-way ANOVA ($P < 0.05$).

Salinity	Inoculum	Populations of MF (×10^3 CFU g^{-1} soil)
0 mM NaCl	CK	0.78±0.40b
	MF	5.22±0.48a
75 mM NaCl	CK	0.56±0.29b
	MF	4.67±0.19a
150 mM NaCl	CK	0.07±0.07b
	MF	2.67±0.37a
225 mM NaCl	CK	0.07±0.07b
	MF	1.53±0.35a
Salinity level (S)		***
Inoculation type (I)		***
S × I		***

Table S3. Operational taxonomic units (OTU) of AM fungi detected in roots (n=24) at four levels salinity (0, 75, 150, and 225 mM NaCl) and inoculum types (CK and MF inoculated treatment).

Salinity	Inoculum	Replicates	#OTUs	#Reads	#AMF Reads	AMF (%)
0 mM NaCl	CK	1	19	22551	22482	99.69
		2	23	24096	24085	99.95
		3	19	24337	24326	99.95
	MF	1	22	23382	23377	99.98
		2	28	23428	23403	99.89
		3	23	22824	22818	99.97
75 mM NaCl	CK	1	29	19303	19295	99.96
		2	25	23536	23532	99.98
		3	28	23601	23597	99.98
	MF	1	28	22113	22111	99.99
		2	22	19475	19475	100.00
		3	33	24169	24160	99.96
150 mM NaCl	CK	1	26	23248	23238	99.96
		2	4	23270	23270	100.00
		3	5	19661	19661	100.00
	MF	1	25	23400	23390	99.96
		2	8	24258	24256	99.99
		3	23	23430	23412	99.92
225 mM NaCl	CK	1	10	24120	24067	99.78
		2	10	22972	22971	100.00
		3	8	24644	24636	99.97
	MF	1	2	23559	23559	100.00
		2	13	24829	24820	99.97
		3	5	23054	22841	99.08

Note: #Reads shows the number of all sequences detected in the sample; #AMF Reads displays the sequences assigned to AM fungi; AMF (%) shows the AM fungi as a percentage of all sequences detected in the sample.

Table S4. *T. longibrachiatum* population density in different salinity levels and inoculum treatments. Values are mean ± SE (n=3). Different asterisk indicate a significant effect of salinity, inoculum and their interaction according to Duncan's multiple range test following significant two-way ANOVA, * $P < 0.05$, ** $P < 0.01$, *** $P < 0.001$ and ns, not significant. Different lowercase letters indicate significant differences among inoculum within each salinity level according to Duncan's multiple range test following significant one-way ANOVA ($P < 0.05$).

Salinity	Inoculum	Populations of MF ($\times 10^3$ CFU g^{-1} soil)
0 mM NaCl	MF	2.7±0.13a
	Gm+MF	2.0±0.12a
75 mM NaCl	MF	3.3±0.07a
	Gm+MF	4.7±0.18a
Salinity level (S)		ns
Inoculation type (I)		ns
S × I		ns

Table S5. Effects of single inoculant and bio-inoculants on the metabolic pathways.

ID	Description	Metabolite Ratio	*P* value	Metabolite ID	Metabolite Name
CK vs. Gm+MF					
ko00520	Amino sugar and nucleotide sugar metabolism	33.33%	0.06	meta_789	N,N'-Diacetylchitobiose
ko01212	Fatty acid metabolism	33.33%	0.06	meta_696	Stearoyl-CoA
ko00460	Cyanoamino acid metabolism	33.33%	0.12	meta_804	Amygdalin
ko01040	Biosynthesis of unsaturated fatty acids	33.33%	0.22	meta_696	Stearoyl-CoA
ko02010	ABC transporters	33.33%	0.37	meta_789	N,N'-Diacetylchitobiose
CK vs. Gm					
ko00460	Cyanoamino acid metabolism	100%	0.04	meta_804	Amygdalin
